# Treatment of Post-Traumatic Diaphyseal and Distal Tibial Bone Defects by Distraction Osteogenesis: A Monocentric Experience

**DOI:** 10.3390/jcm14227933

**Published:** 2025-11-08

**Authors:** Danilo Leonetti, Lorenza Siracusano, Viktor Dietrich Schick, Giovanni Marrara, Leone Larizza, Massimo Brigandì, Angela Alibrandi, Ilaria Sanzarello, Matteo Nanni, Biagio Zampogna

**Affiliations:** 1 Department of Biomedical and Dental Sciences and Morphological and Functional Imaging, Section of Orthopaedic and Trauma Surgery, University of Messina, A.O.U. Policlinico “G. Martino”, 98124 Messina, Italy; lorenza.siracusano@studenti.unime.it (L.S.); viktor.schick@studenti.unime.it (V.D.S.); giovanni.marrara@studenti.unime.it (G.M.); larizzaleone@libero.it (L.L.); massimo.brigandi@unime.it (M.B.); ilaria.sanzarello@unime.it (I.S.); matteo.nanni@unime.it (M.N.); b.zampogna@policlinicocampus.it (B.Z.); 2 Department of Human Pathology of Adults and Developmental Age “G. Barresi”, University of Messina, 98125 Messina, Italy; angela.alibrandi@unime.it; 3 Operative Research Unit of Orthopaedic and Trauma Surgery, Fondazione Policlinico Universitario Campus Bio-Medico, 00128 Rome, Italy; 4 Research Unit of Orthopaedic and Trauma Surgery, Department of Medicine and Surgery, Università Campus Bio-Medico Di Roma, 00128 Rome, Italy

**Keywords:** bone transport, distraction osteogenesis, Ilizarov technique, external fixation, bone defect, tibia

## Abstract

**Background:** Distraction osteogenesis (DO) and the Masquelet technique are currently the preferred treatment options for bone defects larger than 5 cm. **Methods:** Between January 2019 and December 2023, 19 patients were treated with DO for post-traumatic tibial defects at our institution. The results were evaluated using the Association for the Study and Application of the Method of Ilizarov (ASAMI) scoring system. **Results:** Patients’ mean age was 32.42 years. The mean defect size was 4.8 cm (range 3.2–8.1 cm), and the mean external fixation time was 21.31 weeks. Bone union was reached on average after 25.9 weeks. The mean follow-up time was 3.7 years. The mean leg length discrepancy at the final follow-up was 0.83 cm. Using the ASAMI system, the functional results were excellent in eight patients, good in eight, and fair in two, with one case of failure; the bone results were excellent in ten patients, good in six, fair in two, and poor in one. **Conclusions:** DO for the treatment of tibial defects has the potential to yield favourable outcomes, provided that the method is performed correctly. Multicentre prospective studies would allow for more definitive conclusions to be made.

## 1. Introduction

Tibial bone defects represent a significant challenge in orthopaedic surgery for both surgeons and patients. Such defects may arise from severe trauma, infection, or tumour resection and may require repeated surgeries, resulting in protracted treatment times and the potential for complications [1]. The soft tissue envelope covering the tibia is thin, and vascularity in the tibial diaphysis is scarce, increasing the risk of infection and non-union [2,3]. There are a number of reconstructive surgical techniques available, namely autologous bone graft (ABG), vascularised fibular grafting (VFG), distraction osteogenesis (DO), and the induced membrane technique (IMT, Masquelet). The choice of treatment depends on the patient characteristics and the size of the defect. Distraction osteogenesis and the Masquelet technique are currently the preferred treatments for defects larger than 5 cm [4].

Distraction osteogenesis is a surgical procedure that involves the use of an external fixator or an intramedullary nail to transport a segment of bone in a controlled manner to close a bone defect. The technique was first described by Ilizarov, who pioneered its use in cases of non-union with fine wire circular fixation [5]. Its subsequent rise in popularity can be attributed to the development of new fixation devices, including unilateral and spatial frames [4]. The initial step is meticulous preoperative planning, which requires full-length radiographs of the lower extremity and possibly the utilisation of computed tomography. The frame is placed using wires and half-pins. A metaphyseal corticotomy is performed using a drill and an osteotome, thus representing the regenerative site [6]. The resulting free segment is then transported to fill the bone defect with newly generated vital bone until the docking site is reached [7].

According to the traditional Ilizarov technique, the transportation device is typically composed of an external ring fixator with small-diameter transosseous wires, as it has been demonstrated that patients tend to have greater tolerance to small wires. The utilisation of a circumferential device allows for the transportation of bone in a triplanar way to correct deformity and control bone regeneration in axial, rotational, and angular directions [8].

New hybrid techniques have been developed which combine external and internal fixation to reduce the time required for external fixation and decrease the overall healing time.

Distraction osteogenesis is a highly reliable method for the reconstruction of segmental tibial defects, with reported union rates of up to 97% [4,9]. The primary benefit of this method is that it is not constrained by the size of the defect that requires reconstruction [9]. Distraction osteogenesis facilitates early weight-bearing, minimises the risk of additional damage to soft tissues, and enables complete infection eradication by extracting avascular and potentially infected bone components prior to the initiation of the transport process [4,9]. The primary disadvantage associated with this approach is the protracted nature of the reconstruction process, which typically spans a duration of 10–12 months for a defect measuring 10 cm [10].

This has been demonstrated to be a significant source of psychological distress for patients, often resulting in diminished compliance and an increase in complications. Furthermore, distraction osteogenesis carries the risk of fracture of the newly regenerated bone and the potential risk of deformity once the external fixation frame has been removed [11].

At our institution, we currently employ DO as the main treatment option for post-traumatic bone defects. The aim of this paper is to retrospectively analyse the results of a consecutive case series of patients with post-traumatic bone defects treated with distraction osteogenesis.

## 2. Materials and Methods

Following the acquisition of institutional board approval (Prot.98-24), a retrospective analysis of patients afflicted by post-traumatic tibial bone loss treated with distraction osteogenesis at a single institution between January 2019 and December 2023 was conducted. Patients’ data were extracted from the institutional registry. The inclusion criteria for this study comprised patients over the age of 18 years who had sustained high-energy open tibial fractures accompanied by bone loss or subsequent aseptic or septic non-unions, necessitating bone resection. The study population needed to have undergone a minimum of one year of clinical and radiographical follow-up.

Patients who were unable to give consent and underwent distraction osteogenesis due to tumour resection or other non-traumatic conditions were excluded from the study.

In the present study, patients were treated with either the DO by the traditional Ilizarov technique or by a hybrid technique, which incorporated external fixation in conjunction with internal fixation (nailing or plating). In circumstances where no active infection was present and damage to soft tissue was solved at the intended site for internal fixation, the hybrid technique was employed.

The remaining patients were treated using the Ilizarov technique, which involved circular external fixation and bifocal transportation.

The indications for distraction osteogenesis, either with the use of circular external fixation (Ilizarov technique) or with a hybrid technique, were as follows: post-traumatic bone loss, septic non-union, and aseptic non-union.

The surgical intervention was carried out by the senior author (DL) at a single institution.

Treatment for each patient was planned using computed tomography scans. The patient was placed in supine position on a radiolucent table under spinal anaesthesia. Following debridement, the Ilizarov external frame was assembled and a percutaneous corticotomy was performed in the proximal tibia. After a latency period of approximately seven days, the bone segment was transported 1 mm per day in proximo-distal direction until compression of the docking site and callus formation.

**Figure 1:**
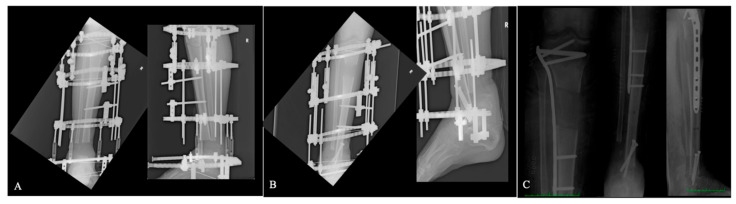
Patient affected by septic non-union treated with the hybrid technique. (**A**) Initial application of circular external fixation. (**B**) Reached docking site and tibiotalar arthrodesis. (**C**) Plating to protect the regenerative site.

However, if there was no active infection after the distraction phase and soft tissue covering was adequate, the hybrid technique was employed and nailing or plating was performed (Figure 1).

Postoperatively, patients were followed-up in the outpatient clinic on a bi-weekly basis, or more frequently in cases of severe soft tissue damage.

The evaluation of functional and radiographic results was conducted in accordance with the ASAMI (Association for the Study and Application of the Method of Ilizarov) scoring system [12]. The following parameters were recorded: number of previous surgeries before DO, time from trauma to DO, external fixation time (EFT), time to bone union, final leg length discrepancy (LLD), and peri- and postoperative complications.

### Statistical Analysis

We performed an a posteriori power analysis, considering the “Bone defect size” as the primary variable. More specifically, we postulated a hypotheses system in which the alternative hypothesis is unidirectional (one-tailed) for one sample, assuming an effect size of 0.60 (deriving from a “bone defect size” with mean = 4.8 and SD = 1.32) and a type I alpha error of 5%. The number of patients being equal to 19 allows us to guarantee the achievement of the minimum power of 80%.

Numerical data were expressed as mean and standard deviation (S.D.), and the categorical variables were expressed as absolute and percentage. The parametric approach was used since the numerical variables were normally distributed, such as verifying them using the Kolmogorov–Smirnov test.

In order to identify possible significant differences between patients according to smoking habit (yes or no), Student’s t-test was applied with reference to the numerical parameters (age, weight, height, BMI, etc.), and the chi-square test (or alternatively the likelihood ratio test or Fisher’s exact test, as appropriate) was applied with reference to the categorical variables (gender, diagnosis, tibial segment, etc.). The same analysis was performed to compare infection (yes or no).

Statistical analyses were performed using IBM SPSS for Windows, Version 22 (Armonk, NY, USA, IBM Corp.).

A *p*-value lower than 0.05 was considered statistically significant.

## 3. Results

Following the analysis of our institutional registry, data from 19 consecutive patients (19 tibiae) were collected. The cohort consisted of 14 males (73.7%) and 5 females (26.3%), with a mean age of 32.42 ± 8.65 years (range 24–54 years). The mean weight of the population was 75.1 kg (range 58–89 kg), and the mean body mass index (BMI) was 25.04 ± 1.54 kg/m^2^ (range 22.1–27.41 kg/m^2^). At the time of surgery, 73.7% of patients were smokers. Demographic details are presented in Table 1.

The data revealed that 57.9% (11 patients) of the population had a diaphyseal tibia defect, while the remaining 42.1% (8 patients) had a distal tibia defect. The left side was affected in 42.1% of cases, and the right side in 57.9% of cases.

The mean number of previous surgical procedures was 2.36 ± 1.25 (range 1–4) (Table 2). A statistically significant difference was observed in the number of previous procedures between the septic and non-septic groups (*p*-value = 0.004).

Post-traumatic bone loss was observed in eight patients (42.1%), while septic non-union was identified in ten patients (52.6%). A single case (5.3%) presented with aseptic non-union.

All cases sustained high-energy trauma, including 11 road traffic accidents (57.9%), 6 falls from height (31.6%), and 2 occupational injuries (10.5%).

Among the ten patients affected by septic non-union, Staphylococcus Aureus was the most frequently isolated pathogen (n = 5), followed by Enterococcus Faecalis and Staphylococcus Epidermidis (n = 2 each).

The following bacteria were isolated just once: methicillin-resistant Staphylococcus Aureus, Enterobacter cloacae complex, and Pseudomonas aeruginosa.

Single cases of infection due to methicillin-resistant *S. aureus*, Enterobacter cloacae complex, and Pseudomonas aeruginosa were also recorded. One mixed bacterial–fungal infection (*S. epidermidis* + *Candida fersififorms*) was detected, and three patients had polymicrobial infections (Table 2).

A statistically significant correlation was observed between smoking and the number of prior surgeries before distraction osteogenesis (*p*-value = 0.029).

Although a higher prevalence of smokers was noted among patients with septic non-union (64.3%), this association did not reach statistical significance (*p*-value = 0.07).

There was no statistically significant correlation between the location of the defect (diaphyseal or distal) and the development of septic non-union (*p*-value = 0.4).

Six patients (31.6%) were treated with the traditional Ilizarov technique (external fixation only); two of these patients underwent arthrodesis of the tibiotalar joint. The remaining thirteen patients (68.4%) were treated using a hybrid technique, involving intramedullary nailing in ten cases (76.9%) and plate fixation in three (23.1%). One of these patients was treated by bone transport over a nail (Table 3).

The mean interval between injury and distraction osteogenesis was 6.1 ± 8.19 months (range 1–36 months). This delay can be attributed to the fact that a significant number of patients affected by non-unions were not diagnosed or treated in a timely manner.

The mean post-traumatic or post-debridement bone defect size was 4.8 ± 1.32 cm (range, 3.2–8.1 cm), with significantly larger defects in the septic non-union group compared with aseptic and bone loss cases (*p*-value = 0.021).

The mean external fixation time was 21.31 ± 5.13 weeks (range 13–29 weeks). The mean follow-up period was 3.7 years (160.1 ± 57.1 weeks; range 68–270 weeks). Bone union was achieved at an average of 25.89 ± 8.34 weeks. The external fixation time and time to bone union were longer in patients with septic non-union than in those without infection. However, no statistically significant differences were observed between the two groups in either case (*p*-value = 0.107; *p*-value = 0.741).

No intraoperative complications were recorded. Postoperative complications related to the DO procedure occurred in three patients (15.78%) and included two pin tract infections and one case of K-wire breakage, which required a re-intervention. One patient underwent a below-the-knee amputation due to sepsis. Another patient exhibited low compliance, resulting in an unsatisfactory (fair) outcome. The mean leg length discrepancy at the final follow-up was 0.83 ± 0.61 cm (range 0.2–2.3 cm) (Table 3).

Finally, according to the ASAMI classification, functional outcomes were excellent in eight patients (42.1%), good in eight (42.1%), fair in two (10.5%), and poor in one (5.3%). Regarding the bone results, we achieved excellent results in ten patients (52.6%), good results in six (31.6%), fair results in two (10.5%), and poor in one patient (5.3%) (Table 4). There were no statistically significant differences in ASAMI scores between groups (*p*-value = 0.27). However, the patient who underwent amputation had septic non-union with a history of heavy smoking and was not compliant.

## 4. Discussion

Distraction osteogenesis was first described by Ilizarov in the 1950s to treat severe bone defects caused by trauma, infection, non-union, or tumour. The traditional technique was based on the use of circular fixation to provide a framework for segmental transport and distraction to allow for osteogenesis and restoration of the defect. According to this technique, the affected bone is resected until a healthy area is reached. Gradual transportation is then performed [6,13]. The success of the method in the 1960s led to the rapid spread of the technique, and it is now the gold standard for segmental defects [14].

Over the years, the technique has evolved, and it is now possible to achieve the same result using intramedullary nailing or hybrid techniques [15]. During the consolidation phase, internal fixation, either nailing or plating, is used to stabilise the segment [4,6]. The hybrid technique has been demonstrated to increase patient comfort, facilitate rehabilitation, and potentially decrease complications [4]. The disadvantages of this procedure include higher costs and the increased risk of deep infection related to the placement of internal devices [4,16].

On the other hand, unilateral frames represent another option with static and dynamic compression capabilities and the use of hydroxyapatite-coated pins [7]; their advantages include the capacity for earlier partial weight-bearing, reduced incidence of pin tract infection, and pin loosening. Additionally, monolateral fixators have been shown to facilitate the management of soft tissue defects because they do not impair the harvesting of a local flap, which is a limitation encountered with ring fixators [1,7].

Other standardised treatments for large bone defects, as mentioned above, are the induced membrane technique (Masquelet), the use of vascularised bone grafts (VBGs), allografts, or bone substitutes [17,18]. A new frontline of care is represented by custom-made titanium implants [19]. However, Ilizarov’s method has proven to be particularly effective in critically sized defects (CSDs), allowing for gradual correction with minimal invasiveness [20,21].

Each of these procedures has its disadvantages. For instance, vascularised bone grafting has been associated with potential complications at the donor site, such as pain, muscle weakness, and sensory alterations and risk of re-fracture up to 20% within the first year [4]. On the other hand, autologous bone grafting has been proven to be ineffective for defects larger than 5 cm due to graft reabsorption.

Consequently, DO and Masquelet are the most commonly used techniques for CSDs [22,23]. Because of the proximity of neurovascular structures, Masquelet and VBGs are often preferred in upper-limb reconstruction to minimise distraction-related neurovascular injury [8]. In addition, Ilizarov’s technique can be used for both extensive bone and soft tissue injuries, as described by Paley et al. [24]. Nevertheless, external fixation is related to discomfort for the patient and a variety of possible complications. Immediate complications include neurovascular damage during pin insertion or corticotomy. Early complications are related to bleeding, pin tract infection, hardware failure, sensory abnormalities due to nerves stretching, compartment syndrome, and deep vein thrombosis. Late complications include osteomyelitis, premature union at the regenerative site, or non-union [25,26].

The current literature reports favourable outcomes with both the induced membrane technique and DO for the treatment of CSDs, but no universal consensus exists regarding the optimal approach [27]. A recent systematic review and meta-analysis by Wakefield et al. has shown that there is no clinical difference in outcomes between Ilizarov and IMT in managing tibial septic non-unions [9]. Conversely, Benulic et al. observed higher union rates (92–100%) and lower infection rates (0–4%) in the DO group compared with IMT (union 42–100%; infection 12–43%) in regard to high-grade open tibia fractures, thus reaching statistical significance in favour of DO [23]. Khaled et al., instead, proposed a combined DO-IMT approach to merge the advantages of both techniques while avoiding their respective drawbacks, reporting good outcomes in more than 90% of patients [20].

In the present study, 18 of 19 patients (94.7%) achieved bone union, consistent with published data [9]. Bone union was achieved in all patients, regardless of their initial diagnosis. We recorded a difference in the time taken for bone union between septic and non-septic patients, in favour of the non-septic patients. However, this difference was not statistically significant, probably also because there was no statistical significance according to bone size defect.

Haase et al. reported a similar rate of bone union but increased EFT and number of previous procedures in the septic group for non-union in long bones when compared to the aseptic cohort [28].

Two patients (10.1%) developed pin tract infection. This is much lower than the 60% reported by Wakefield et al. but higher than the rates reported by Benulic et al. [9,23]. Our group previously reported a 25% incidence of pin tract infection in a recent systematic review [21]. To minimise the risk of pin tract infection, it is recommended that a pin sleeve is used during the insertion process. In addition, screws should be inserted perpendicularly through both cortices, aligned with the medullary canal, to prevent loosening and infection [3].

None of the patients in this cohort presented a residual leg length discrepancy greater than 2.5 cm; therefore, this was not classified as a postoperative complication.

Overall, 4 complications occurred in 19 patients, meaning that there was an average of 0.21 complications per patient. Of these, one was a major complication (amputation), and three were minor complications (two pin tract infections and K-wire breakage).

Feng et al., in their series of 199 tibial bone defects treated with Ilizarov bone transport, reported an average of 1.04 minor and 0.48 major complications per patient [29].

Complications were observed in patients exhibiting diaphyseal defects. However, it remains uncertain whether the location of the defect may be a contributing factor to a more unfavourable outcome.

Liu et al. reported a higher rate of excellent and good bone results in the diaphyseal group, higher than those in the proximal and distal groups. In contrast, the findings indicated that the proximal group demonstrated the highest rate of excellent and good function results. Distal tibial bone transport complications were found to be more severe and occurred at a higher rate than in other bone sites [3].

A recent meta-analysis by Tian et al. evaluated the association between influencing factors and non-union of tibial fractures. They identified 15 factors that significantly influence fracture union, including diaphyseal and distal tibial over the proximal segment [30].

A systematic review published in 2025 showed satisfactory bone results (excellent + good) according to the ASAMI scoring system in 87.3% of cases and satisfactory functional results (excellent + good) in 88% of patients treated with DO for post-traumatic tibial defects [21].

In the present cohort, satisfactory outcomes were achieved in 84.2% of patients for both bone and functional results, corroborating findings from the current literature.

The present study has several limitations. First of all, the retrospective design without randomisation or a comparison group is a limitation. Moreover, the sample size is small, and data from only a single institution are presented. Additionally, the population was heterogeneous as we did not distinguish between bone loss and aseptic or septic non-unions. Since all included patients were treated by DO, there is no direct comparison with other treatment techniques.

## 5. Conclusions

The present study achieved satisfactory (good–excellent) functional results in 84.21% of cases, which is consistent with the existing literature.

The DO technique for the treatment of tibial defects has the potential to yield favourable to excellent outcomes, provided that the method is performed correctly with compliance from the patients.

In our opinion, further multicentre prospective studies are needed, including the Masquelet technique and 3D-printed custom-made titanium implants, to allow for more definitive conclusions to be drawn.

## Figures and Tables

**Table 1:** Demographic data.

	n° (%)	Age (Years)	Weight (kg)	Height (m)	BMI (kg/m^2^)	Smoking
Whole	19 (100%)	32.42	75.1	1.73	25.04	14 (73.7%)
Male	14 (73.7%)	34.28	78.1	1.76	26.3	12 (63.2%)
Female	5 (26.3%)	27.2	66.3	1.64	24.7	2 (10.5%)

BMI: body mass index.

**Table 2:** Baseline data of study patients.

N°	Mechanism of Injury	Injury Side	Location of Bone Defect	Bone Defect Size (cm)	Previous Surgeries (n°)	Time from Trauma to DO (Month)	Bacterial Culture
1	Fall from height	Left	Diaphyseal	4.7	3	14	EF
2	Road accident	Right	Diaphyseal	4	1	1	/
3	Fall from height	Right	Diaphyseal	5.2	4	12	SE + ECC
4	Road accident	Right	Distal	4.6	2	2	/
5	Workplace injury	Left	Diaphyseal	5.2	2	2	/
6	Road accident	Left	Diaphyseal	3.2	1	1	/
7	Fall from height	Left	Distal	4.7	2	3	PA
8	Road accident	Left	Distal	5.1	2	7	SA
9	Road accident	Right	Distal	3.4	1	1	/
10	Fall from height	Right	Distal	6.3	3	36	EF + SA
11	Road accident	Right	Distal	4.1	2	4	SE + CF
12	Road accident	Right	Diaphyseal	3.2	3	6	SA
13	Road accident	Left	Diaphyseal	4.2	4	2	/
14	Fall from height	Left	Diaphyseal	7.2	3	10	SA
15	Road accident	Right	Diaphyseal	8.1	5	3	MRSA
16	Workplace injury	Right	Distal	3.4	1	2	/
17	Road accident	Left	Distal	4.1	1	2	/
18	Fall from height	Right	Diaphyseal	5.8	3	6	SA
19	Road accident	Right	Diaphyseal	4.8	3	2	/
Average				4.8 ± 1.32	2.47	6.1 ± 8.1	

EF: Enterococcus Faecalis; MRSA: methicillin-resistant Staphylococcus Aureus; SE: Staphylococcus Epidermidis; ECC: Enterobacter Cloacae Complex; SA: Staphylococcus Aureus; PA: Pseudomonas Aeruginosa; CF: Candida Fersififorms; DO: Distraction Osteogenesis.

**Table 3:** Characteristics of DO treatment, follow-up, and complications.

N°	Surgery	EFT (Weeks)	Time to Bone Union (Weeks)	Surgical Complications	Post-Surgery Complications	FU (Weeks)	LLD (cm)
1	Ilizarov	28	N/A	/	AKA	147	N/A
2	Hybrid (Ilizarov + nailing)	17	25	/	Pin tract infection	180	1
3	Hybrid (Ilizarov + nailing)	25	31	/	/	130	2.3
4	Hybrid (Ilizarov + nailing)	20	26	/	/	222	0.2
5	Ilizarov	26	33	/	/	243	0.3
6	Hybrid (Ilizarov + plating)	15	20	/	/	135	0.3
7	Hybrid (Ilizarov + nailing)	17	26	/	/	205	1.2
8	Hybrid (Ilizarov + nailing)	21	28	/	/	152	1
9	Hybrid (Ilizarov + nailing)	19	25	/	/	170	1.1
10	Ilizarov + Arthrodesis at the Docking site	28	36	/	/	203	0.2
11	Ilizarov + Arthrodesis at the Docking site	17	21	/	K wires breakage	203	1.4
12	Hybrid (Ilizarov + nailing)	13	19	/	/	78	1.7
13	Hybrid (Ilizarov + nailing)	16	21	/	/	270	1.2
14	Hybrid (Ilizarov + nailing)	29	35	/	/	97	1.4
15	Hybrid (Ilizarov + plating)	27	36	/	/	182	0.3
16	Hybrid (Ilizarov + plating)	16	22	/	/	68	0.2
17	Hybrid (Ilizarov + nailing)	21	25	/	/	95	0.7
18	Ilizarov	26	33	/	Pin tract infection	174	0.5
19	Ilizarov	24	30	/	/	197	0.8
Average		21.31 ± 5.13	25.89 ± 8.34			160.1 ± 57.1	0.83 ± 0.61

EFT: external fixation time; FU: follow-up; LLD: leg length discrepancy; AKA: above-the-knee amputation.

**Table 4:** ASAMI results at final follow-up.

N°	Bone Results ASAMI	Functional Results ASAMI
1	POOR	FAILURE
2	FAIR	FAIR
3	FAIR	FAIR
4	EXCELLENT	EXCELLENT
5	EXCELLENT	EXCELLENT
6	GOOD	EXCELLENT
7	GOOD	GOOD
8	EXCELLENT	EXCELLENT
9	EXCELLENT	EXCELLENT
10	EXCELLENT	EXCELLENT
11	GOOD	GOOD
12	EXCELLENT	GOOD
13	EXCELLENT	GOOD
14	GOOD	EXCELLENT
15	GOOD	EXCELLENT
16	EXCELLENT	GOOD
17	GOOD	GOOD
18	EXCELLENT	GOOD
19	EXCELLENT	GOOD

## Data Availability

The datasets generated and/or analysed during the current study are available from the corresponding author upon reasonable request.
